# Exploring the biomechanical responses of human cupula by numerical analysis of temperature experiments

**DOI:** 10.1038/s41598-021-87730-w

**Published:** 2021-04-15

**Authors:** Xiang Wu, Shen Yu, Shuang Shen, Wenlong Liu

**Affiliations:** 1 School of Information and Communication Engineering, Dalian University of Technology, Dalian, 116024 China; 2 State Key Laboratory of Structural Analysis for Industrial Equipment, Dalian University of Technology, Dalian, 116024 China; 3 School of Rehabilitation Medicine, Binzhou Medical University, Yantai, 264003 China

**Keywords:** Biomedical engineering, Computational science, Computational biophysics

## Abstract

The vestibular receptor of cupula acts an important role in maintaining body balance. However, the cupula buried in the semicircular canals (SCCs) will be destroyed if it is detached from the relevant environment. The mechanical properties of human cupula still remain ambiguous. In this paper, we explored the cupula responses changing with temperature by experiments and numerical simulation of SCCs model. We obtained 3 volunteers’ nystagmus induced by constant angular acceleration when the temperature of volunteers’ SCCs was 36 °C and 37 °C respectively. The slow-phase velocity of 3 volunteers decreased by approximately 3°/s when the temperature of SCCs reduced by 1 °C, which corresponded to the reduction of cupula deformation by 0.3–0.8 μm in the numerical model. Furthermore, we investigated the effects of the variation of endolymphatic properties induced by temperature reduction on cupula deformation through numerical simulation. We found that the decrease of cupula deformation was not caused by the change of endolymphatic properties, but probably by the increase of cupula’s elastic modulus. With the temperature reducing by 1 °C, the cupula’s elastic modulus may increase by 6–20%, suggesting that the stiffness of cupula is enhanced. This exploration of temperature characteristic of human cupula promotes the research of alleviating vestibular diseases.

## Introduction

**Figure 1:**
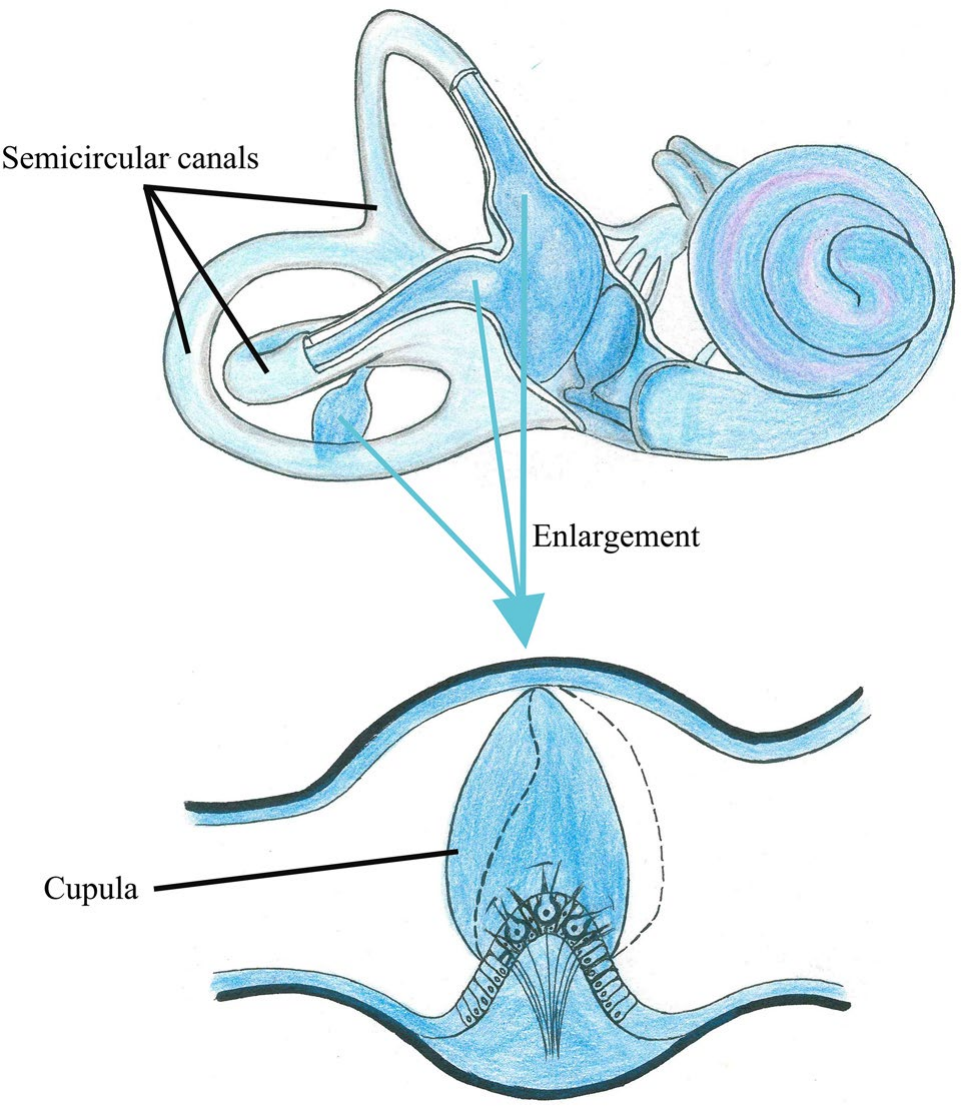
Human SCCs in the vestibular system. Three mutually orthogonal SCCs include utricle, anterior semicircular canal (AC), horizontal semicircular canal (HC), posterior semicircular canal (PC), and 3 cupulae.

The semicircular canals (SCCs) in the vestibular system detect the angular motion of the head^1^. Three SCCs are approximately mutually orthogonal, each of which terminal dilated cavity contains a gelatinous structure called cupula (see Fig. 1). When the head experiences angular motion, the endolymph filled in the SCCs interacts with the cupula due to the inertia. Then, the cupula and the embedded hair cell bundles are deflected, transmitting the neural signals to the brain and inducing the nystagmus to maintain visual stability [the vestibulo-ocular reflex (VOR)]^2,3^. The cupula acts a crucial role in the maintenance of body balance. However, the SCCs are small and complex structures buried in the inner ear. Moreover, the physical properties of cupula will be changed if it is detached from the relevant environment of the SCCs. Thus, it is inappropriate to thoroughly elucidate the material properties and mechanical behaviors of the cupula by dissecting human SCCs. The vestibular diseases involved in the cupula are important factors inducing vertigo, such as motion sickness, benign paroxysmal positional vertigo, and Meniere’s disease, which cause serious disturbance to people's daily life. Some of the vestibular diseases can be induced by excessive stimulation, resulting in large deformation of the cupula. The cupula deformation will be effectively reduced if the cupular stiffness is increased under the condition of same rotational stimulus for SCCs. Then, the vestibular diseases can be appropriately alleviated by increasing the cupular stiffness. Clarifying the mechanical properties of cupula is helpful to find the method to increase the cupular stiffness. Hence, the research of alleviating vestibular diseases will be promoted effectively if the mechanical properties of the cupula are clarified.

In the present studies, McHenry and van Netten^4^ experimentally measured the cupular Young's modulus of zebrafish. These experiments on animals provide a useful basis for researching the mechanical properties of human cupula. However, the anatomic methods used for animal are not suitable for humans. The current technology is not appropriate to directly measure the cupula in human SCCs. Hence, some researchers studied the mechanical behavior of the cupula in SCCs by establishing mathematical or numerical model^2,5–10^. For instance, Selva et al.^5^ built a finite-element model of the cupula and estimated Young’s modulus of human cupula. The cupula is considered as a material of collagen gel because it is a gelatinous flexible structure containing collagen connective tissue fibers^5,11,12^. Collagen is an important factor affecting the stiffness of cupula, and the elastic modulus of collagen gel^11,13^. With the rise of collagen concentration, the elastic modulus of collagen gel increases^13,14^. Furthermore, the variation of the elastic modulus of different concentration in collagen gel changes diversely with temperature^14^. The investigation of the cupula’s elastic modulus changing with temperature may identify the concentration of collagen gel with similar temperature characteristics to the cupula, which is also helpful to determine the approximate range of collagen concentration in the cupula. It plays an important role in clarifying the mechanical properties of cupula and further researching the treatment for vestibular diseases.

Based on the principle of VOR, the cupula is deflected and the physiological response of nystagmus is induced when the SCCs experience angular motion. The slow-phase velocity (SPV) of nystagmus is an external characteristic of cupula deformation, which rises with the cupula deformation increasing^15^. Additionally, the temperature of the segment of horizontal semicircular canal closest to the temporal bone can be reduced by 1 °C when the external auditory canal is provided irrigation of cold air or water that is 7 °C below nominal body temperature (37 °C)^16^. The aims of this study was to quantitatively explore the effects of reducing 1 °C in SCCs on cupula responses by the VOR experiment and numerical simulation of SCCs model. Firstly, 3 volunteers’ nystagmus under the condition of SCCs’ temperature at 36 °C and 37 °C respectively was induced by constant angular acceleration in the VOR experiment. We compared the SPV of volunteers’ nystagmus when their SCCs’ temperature was 36 °C and 37 °C, respectively. Then, the variation of cupula deformation caused by temperature reduction was quantitatively identified in the numerical model of SCCs. Moreover, we explained that the change of the cupula’s elastic modulus might be the dominant factor affecting cupula deformation by describing intricate fluid–structure interaction in the numerical model of SCCs. Finally, we quantitatively investigated the variation of the cupula’s elastic modulus if it was caused by the temperature reduction of 1 °C in SCCs.

## Results

### Volunteers’ SPV

**Figure 2:**
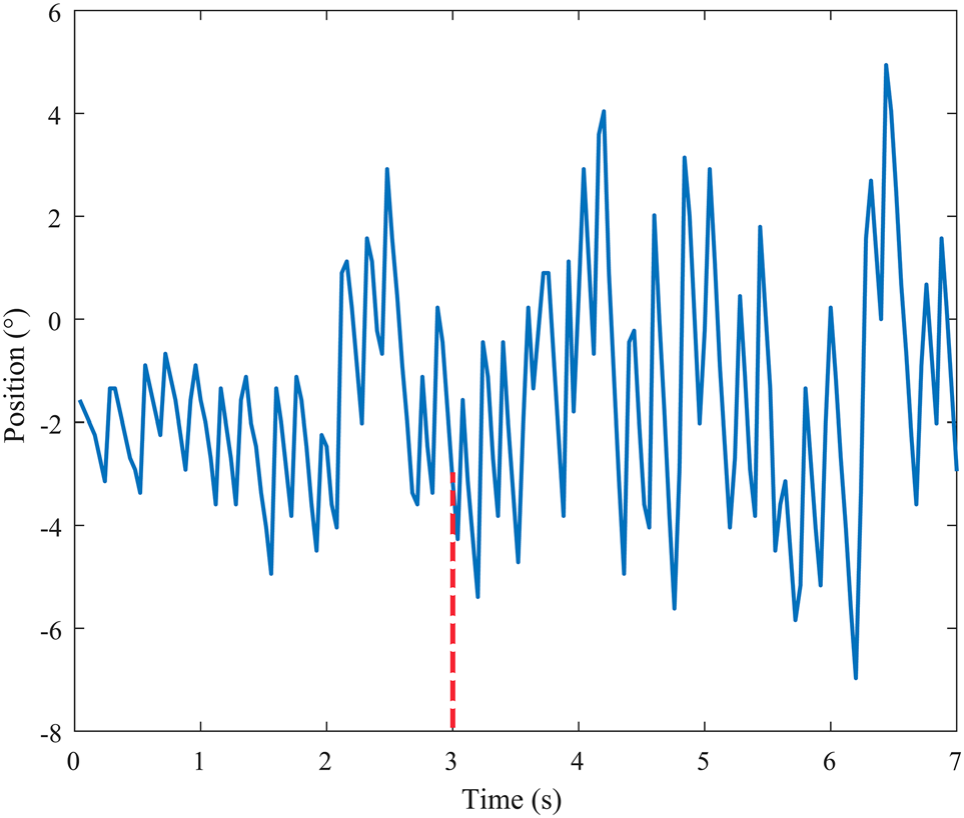
The horizontal nystagmus of a volunteer caused by constant rotational acceleration of 30°/s^2^ during 0–7 s. The horizontal SPV of the volunteer tended to be approximately stable after 3 s.

Figure 2 shows the trajectory of a volunteer’s nystagmus when the temperature of SCCs was 37 °C. The trajectory with the negative slope represents the slow phases of nystagmus. We observed that the volunteer’s SPV gradually increased during 0–3 s, and then tended to be approximately stable. This phenomenon was similar to the result of Bockisch et al. reported^17^. Then, we discarded the volunteers’ SPV in the first 3 s and calculated the average of the SPV in the last 4 s (see Table 1). Table 1 shows the average of the SPV of 3 volunteers’ horizontal nystagmus when the temperature of SCCs was 36 °C (the estimated temperature in this paper) and 37 °C respectively under the constant angular acceleration of 30°/s^2^. When the temperature of SCCs was 36 °C, the volunteers’ SPV was less than that of the SCCs at 37 °C. The results revealed that the volunteers’ SPV induced by constant angular acceleration of 30°/s^2^ was reduced by approximately 3°/s with the temperature of SCCs reducing by 1 °C, which indicated that the cupula deformation decreased. In addition, we found differences in SPV for volunteers when they were simulated by same angular acceleration. The variation of SPV was also different for volunteers when the temperature of SCCs reduced. These phenomena may be caused by individual differences.

**Table 1:** The SPV of 3 volunteers at SCCs’ temperature of 36 °C and 37 °C, respectively. Temperature in the table represents the temperature of SCCs. The unit of SPV is °/s.

Temperature	Nystagmus SPV: mean (standard deviation)	
	36 °C	37 °C
First volunteer	28.92 (3.32)	32.08 (1.55)
Second volunteer	28.27 (2.43)	31.67 (1.31)
Third volunteer	26.36 (3.49)	29.17 (1.85)

### Responses of SCCs

As the temperature of SCCs reduced by 1 °C, the volunteers’ SPV decreased by approximately 3°/s. According to the relationship between cupula deformation and SPV, the SPV rises slowly with the cupula deformation increasing where approximate 0.1–0.2667 μm of the maximal cupula deformation can induce 1°/s of horizontal nystagmus SPV considering individual differences^15^. When the SPV decreased by 3°/s, the cupula deformation could be considered as a linear change with the SPV based on the previous studies^2,18^. Meanwhile, considering that the SPV of volunteers was different due to individual differences, the maximal cupula deformation in the numerical model of SCCs reduced by 0.3–0.8 μm when the SPV decreased by 3°/s. In order to explain the reason for the decrease of cupula deformation, we quantitatively investigated the effects of the changes of endolymphatic properties including the density and viscosity on cupula deformation due to the temperature reduction of SCCs. The transcupular pressure, cupula deformation and strain was compared with the same elastic modulus of cupula (5 Pa) when the temperature of SCCs was 36 °C and 37 °C respectively.

**Figure 3:**
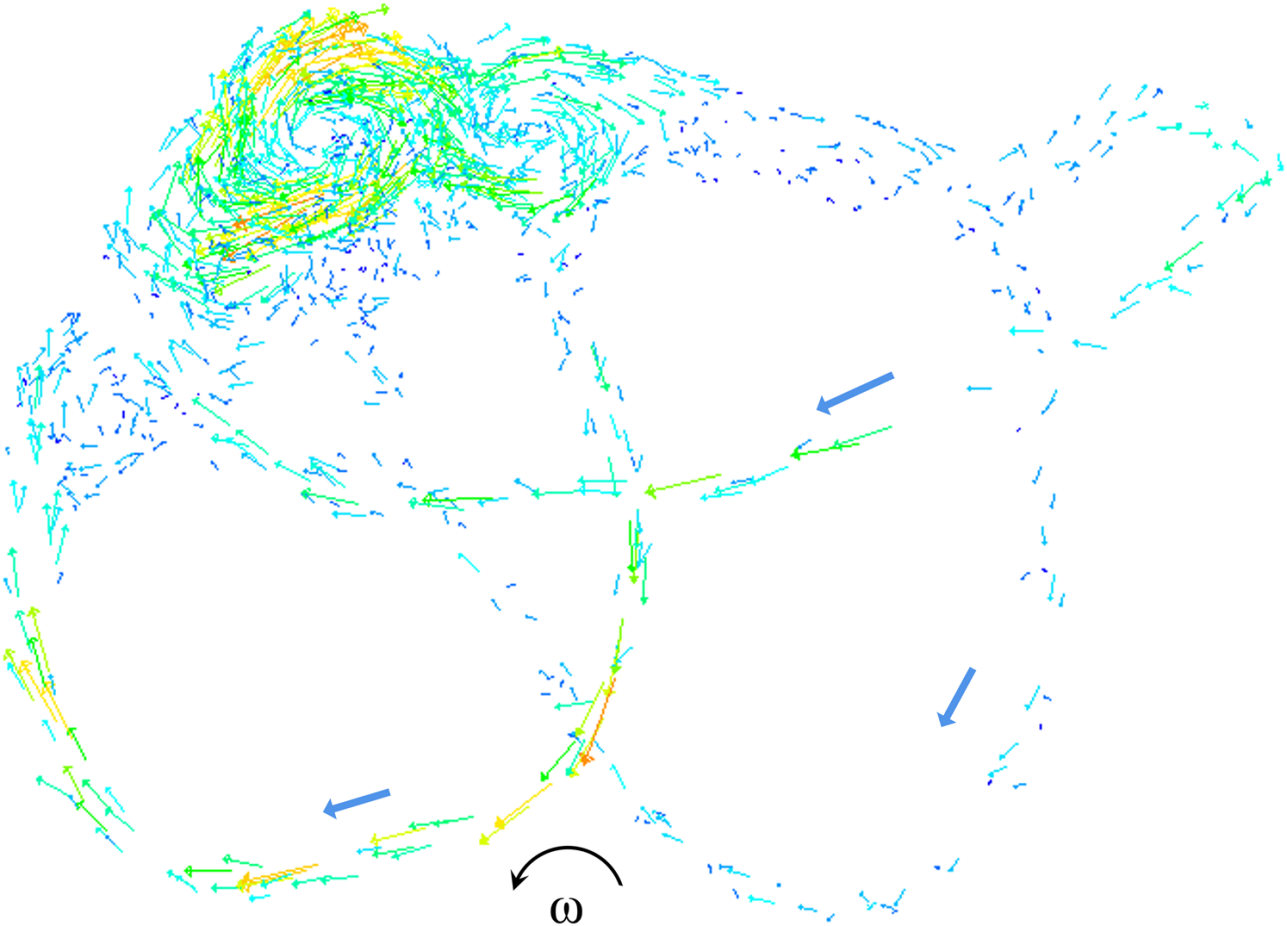
Schematic drawing of the relative movement of endolymph in SCCs at time = 0.1 s. SCCs are rotated with anticlockwise acceleration.

**Figure 4:**
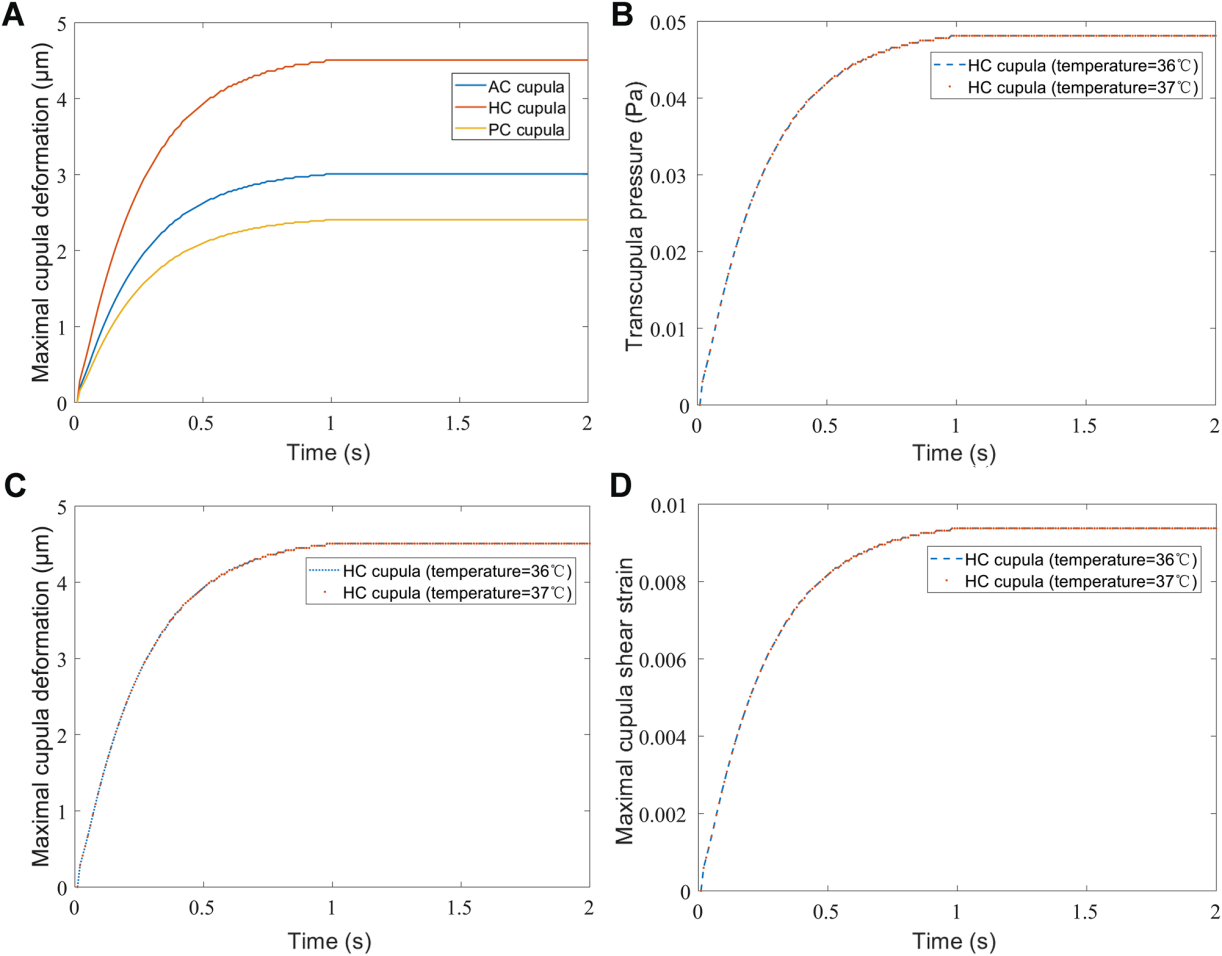
(**A**) The maximal cupula deformation changing with time. (**B**) The maximal transcupular pressure changing with time under condition of 36 °C and 37 °C, respectively. (**C**) The maximal cupula deformation changing with time under condition of 36 °C and 37 °C, respectively. (**D**) The maximal cupula shear strain changing with time under condition of 36 °C and 37 °C, respectively.

**Figure 5:**
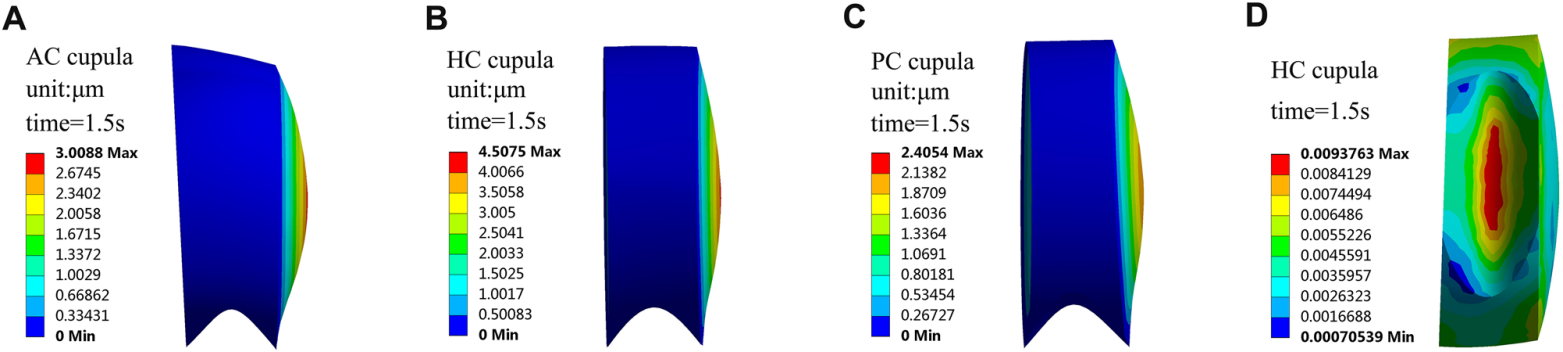
Cupula responses at time = 1.5 s. (**A**) The cupula deformation in AC at 1.5 s. (**B**) The cupula deformation in HC at 1.5 s. **(C)** The cupula deformation in PC at 1.5 s. (**D**) The cupula shear strain in HC at 1.5 s.

When the SCCs rotate with the head, the endolymph is pulled by SCCs due to the action of inertia. The endolymphatic absolute velocity has similar trend with the angular velocity of SCCs. The relative velocity of endolymph at 0.1 s is shown in Fig. 3. There are vortexes in the utricle and tube flow in the narrow duct by observing the relative flow of the endolymph. The direction of the vortexes in the utricle is opposite to that of head angular acceleration. The cupula deformation is induced by the transcupular endolymph pressure^19^. Three cupular deformation at the temperature of 37 °C is shown in Figs. 4A, 5A–C. The maximal cupula deformation increases with the angular velocity of SCCs during 0–1 s. After 1 s, the cupula deformation becomes approximately stable because the force on the cupula induced by the endolymphatic pressure is equal to the elastic force of the cupula. The maximal cupula deformation in HC is greater than that in AC and PC because AC and PC are less stimulated in perceiving angular motion of the head. Besides, sensory cells are distributed on the crista surface of which the shear strain triggers the activation of sensory hair bundles. The SPV depends on the cupula shear strain at the crista surface. Then, we explored the HC cupula shear strain (see Fig. 5D). The result reveals that the cupula shear strain at the center of crista surface is the largest.

When the SCCs are stimulated by the same angular acceleration at 36 °C and 37 °C respectively, the maximal transcupular pressure, cupula deformation, and cupula shear strain in HC changing with time are shown in Fig. 4B–D. The differences between the maximal transcupular pressure, cupula deformation, and cupula shear strain under condition of 36 °C and 37 °C are not significant (see Table 2), indicating that the changes of the endolymphatic density and viscosity caused by temperature reduction have no effects on the cupula deformation and strain. The cupula is a structure of collagen gel whose elastic modulus is sensitive to the change of temperature. When the temperature of SCCs reduced by 1 °C, the variation of the cupula’s elastic modulus might be the dominant factor affecting the cupula deformation. Furthermore, the cupula was considered as a linearly elastic material due to its small deformation^7^. Thus, the maximal cupula deformation reduced by 0.3–0.8 μm considering the individual differences of the volunteers when the temperature of SCCs decreased by 1 °C. The elastic modulus of cupula increased by 0.3–1 Pa, and the percentage increased by 6–20%, which could induce the corresponding reduction of cupula deformation.

**Table 2:** The maximal transcupular pressure, cupula deformation, and cupula shear strain under condition of 36 °C and 37 °C respectively. These numerical results ignore the effect of temperature change on the cupula’s elastic modulus.

Temperature	36 °C	37 °C
Transcupular pressure (Pa)	0.04814	0.04813
Cupula deformation (μm)	4.509	4.508
Cupula shear strain	0.009379	0.009376

## Discussion

Hair cell bundles are located on the crista surface in the cupula. The cupula shear strain at the crista surface induces the activation of the hair cell bundles that transmit the neural signals to the brain and trigger the corresponding nystagmus. Although the SPV depends on the cupula shear strain, the SPV is also dependent on the cupula deformation because the cupula deformation and strain are correlated^15^. Thus, the SPV can be the external characteristic of cupula deformation. Besides, the maximal cupula shear strain is at the center of crista surface (see Fig. 5D), which is consistent with the descriptions by Goyens et al.^9^. There are the most sensitive nerves in the center of crista surface^20^, increasing the sensitivity of detecting angular motion. When the SCCs experience horizontal rotation, the HC is more stimulated than other SCCs. Then, the HC cupula deformation is the greatest. The hair cell bundles embedded in the HC cupula become active, transmitting the neural signals to the brain and triggering horizontal nystagmus, which is consistent with the results of VOR experiment.

When the volunteers’ head was rotated with the constant angular acceleration of 30°/s^2^, each volunteer’s SPV at the SCCs’ temperature of 37 °C was greater than that at 36 °C. Considering that SPV is an external characteristic of cupula deformation, SPV rises with the cupula deformation increasing^15^. Hence, temperature reduction of SCCs induced the decrease of cupula deformation which resulted in the decrease of SPV. The elastic modulus of cupula, the density and the viscosity of endolymph changed after the temperature of SCCs dropped by 1 °C. However, the variation of endolymphatic density and viscosity caused by the temperature reduction had not the effects on the transcupular pressure that caused cupula deformation. Additionally, the cupula is considered as a material of collagen gel because of its a gelatinous flexible structure containing collagen connective tissue fibers^5,11,12^. The elastic modulus of collagen gel is easily affected by temperature. Thus, the decrease of cupula deformation was not induced by the variation of the endolymphatic density and viscosity, but probably by the increase of the cupula’s elastic modulus. The increase of cupula’s elastic modulus induced the increase of cupula’s stiffness with the temperature reducing by 1 °C, which led to the decrease of cupula deformation. The neural signals generated by the hair cell bundles embedded in cupula were reduced, resulting in the decrease of volunteers’ SPV. When the SPV of volunteers decreased by approximately 3°/s, the cupula deformation in the numerical model of SCCs reduced by approximately 0.3–0.8 μm. Some studies suggested that the SPV of nystagmus was proportional to the cupula deformation^2,18^. Although the SPV increases nonlinear with the cupula deformation rising in our previous work^15^, the SPV can be considered to be proportional to the cupula deformation when the variation of SPV is very small. Besides, the real elastic modulus of human cupula remains ambiguous because the present technology can not directly measure it. In order to investigate the change of percentage for the cupula’s elastic modulus, we set the elastic modulus of cupula as 5 Pa in numerical simulation, which was estimated by Selva et al.^5^. The variation of percentage for the cupula’s elastic modulus increased by 6–20%, which was not affected by the specific value of elastic modulus. The reason was that the cupula was considered to be a linearly elastic material, and then the cupula’s elastic modulus varied linearly with the SPV when the temperature of SCCs changed with the same rotational stimulus. Additionally, the cupula is considered as a soft structure of collagen gel. The elastic modulus of 0 to 3 mg/ml collagen gel is the same order of magnitude as that of cupula^5,13,14^. When the temperature reduces from 37 to 36 °C, the elastic modulus of 2 mg/ml collagen gel increases by approximately 6.8%^14^, which is in the percentage increment range of the cupula’s elastic modulus. The cupula has the similar properties to 2 mg/ml collagen gel, suggesting that the collagen concentration in the cupula may be around 2 mg/ml. In fact, due to individual differences, the properties of cupula for different people may be diverse, including the elastic modulus, collagen concentration, and temperature characteristics. The differences in mechanical properties of the cupula for people with normal vestibular function may be within reasonable ranges.

The cupula, the vestibular receptor, is associated with the vestibular diseases, such as motion sickness, benign paroxysmal positional vertigo and Meniere’s disease. When the temperature of SCCs decreases from 37 to 36 °C, the elastic modulus of the cupula may increase as well as the stiffness, which improves the ability of cupular anti-deformation. Compared with nominal body temperature (37 °C) under the same acceleration stimulation, the cupula had smaller deformation after reducing its temperature. Then, the deflection of hair cell bundles embedded in the cupula became smaller than normal. As a result, the nervous signals generated by hair bundles were weakened. Thus, some vestibular diseases can be alleviated appropriately. In this study, a novel method was provided to explore the elastic modulus of human cupula by VOR experiment and numerical simulation of SCCs model, which might promote the research of relieving vestibular diseases. Moreover, as the cupula is considered to be the material of collagen gel, it is helpful to realize the mechanical properties of the cupula by determining the collagen concentration in the cupula. In this paper, we found that the collagen concentration in the human cupula might be around 2 mg/ml. The elastic modulus and temperature characteristics of 2 mg/ml collagen gel are close to those of the cupula, indicating that they are likely to have more similar mechanical properties. In future studies, the exploration and verification of similar properties for both cupula and collagen gel will act a crucial role in the research of mechanical properties of human cupula, which benefit the study of clinical treatment for vestibular diseases.

## Conclusions

We performed the VOR experiments for 3 volunteers under the constant angular acceleration of 30°/s^2^ when the temperature of SCCs was 36 °C and 37 °C respectively. Three volunteers’ SPV decreased by approximately 3°/s with the temperature of SCCs reducing by 1 °C. In the numerical model of SCCs we constructed, the cupula deformation decreased by approximately 0.3–0.8 μm. Besides, we explored the effects of the change of the endolymphatic density and viscosity induced by temperature reduction on cupula deformation. We found that the cupula deformation almost remained unchanged by constructing numerical model to describe intricate fluid–structure interaction in SCCs. The increase of the cupula’s elastic modulus may be the dominant factor for the decrease of cupula deformation when the temperature of SCCs is reduced by 1 °C. Considering individual differences, the elastic modulus of the cupula may be increased by 6–20% with the temperature of SCCs reducing by 1 °C. The elastic modulus of the cupula increases as well as the stiffness, improving the ability of cupular anti-deformation, which promotes the research of clinical treatment for vestibular diseases.

## Materials and methods

### Volunteers and equipment in the VOR experiment

Three volunteers participated in the VOR experiment, and provided written informed consent to their participation prior to the experiment. Besides, the statement that all volunteers agreed to publish their identifiable information or images in an online open-access publication was included in the written informed consent. They were informed of the experimental procedures, and were allowed to stop the experiment at any time. All of them had normal vestibular function without history of vestibular or ocular diseases. The experiments were approved by the ethics committee of Dalian university of technology, and were in line with the principles of the Declaration of Helsinki; the registration number was 2020-077.

**Figure 6:**
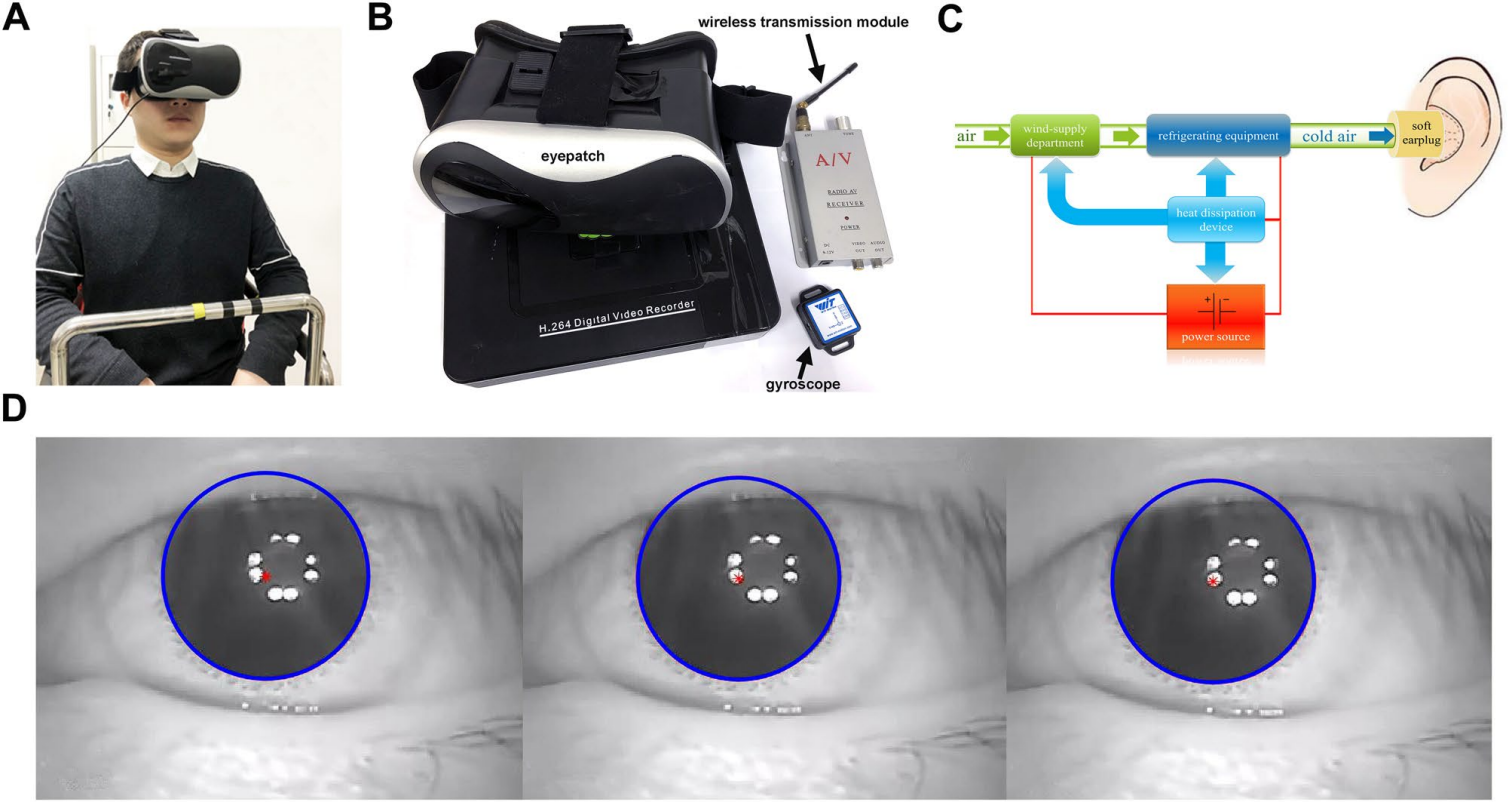
(**A**) The volunteer sitting on the chair. (**B**) The experimental equipment used for recording volunteers’ eye movement. (**C**) Schematic drawing of the refrigerating device for volunteers. (**D**) Locating the center of the pupil in the volunteers’ eye movement.

As shown in Fig. 6A, a volunteer was sitting on the rotatable chair. The experimental equipment including eyepatch, gyroscope, wireless transmission module and battery was used for recording eye movement (see Fig. 6B). A small infrared camera was embedded in the eyepatch to monitor the left eye. Besides, the gyroscope fixed on the right side of the eyepatch could measure instantaneous angular velocity; the sampling frequency of the gyroscope was up to 200 Hz, and we used 50 Hz. There was no relative movement between the eyepatch and the head when the volunteers wearing the eyepatch sensed the angular motion of head. Then, the gyroscope could real-time monitor the angular velocity of the volunteers’ head. The wireless transmission module included transmitter and receiver module; the transmitter module connecting to the infrared camera transmitted the video signals of recording eye movement to the receiver module which was connected to the computer; the eye movement videos were stored on the computer. Additionally, the headrest was fixed to the chair to reduce the relative movement of the volunteers' heads during rotation, and the volunteers were secured to the chair with safety belts. Figure 6C shows the working process of the refrigerating device which has the function of producing cold air and transmitting it to the external auditory canal of the volunteers. The refrigerating device consisted of five modules including power source, wind-supply department, refrigerating equipment, heat dissipation device, and air transmission apparatus. Lithium battery was selected as power source, which supplied appropriate electricity for other modules. The flowing air was generated by the wind-supply department, which was transmitted to the refrigerating equipment through the air transmission apparatus. The refrigerating equipment produced cold air depending on three semiconductor chilling plates in it. There were several air ducts constituting the air transmission apparatus. The terminal of the air ducts were inserted into the soft earplugs which were probed into the ear to transmit the cold air (below 26 °C) to the external auditory canal. The temperature of the segment of horizontal semicircular canal closest to the temporal bone can be reduced by 1 °C when the external auditory canal is provided irrigation of cold air or water that is 7 °C below nominal body temperature (37 °C)^16^. Then, the temperature of SCCs could be reduced to 36 °C when the external auditory canal of volunteers was continuously irrigating by cold air. Besides, the earplugs had multiple air holes, which ensured that the air flowed out of the earplug air holes normally without harm for the volunteers. The heat generated by the electronic equipment was dissipated by the cyclic water in the module of the heat dissipation device.

### Experimental procedure

**Figure 7:**
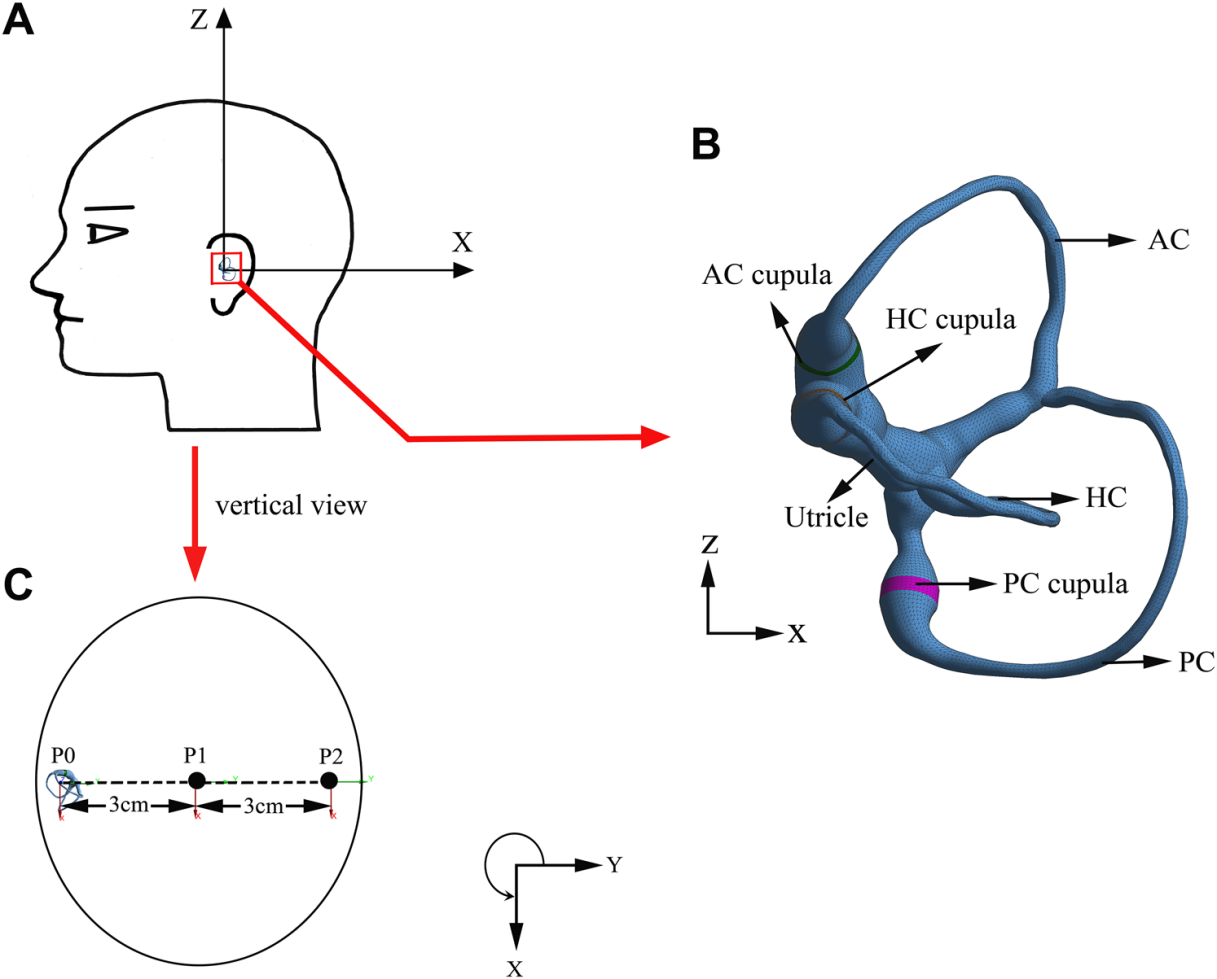
(**A**) Schematic drawing of the SCCs in the left ear. (**B**) The reconstructed numerical model of left SCCs. The model includes utricle, AC, HC, PC, and 3 cupulae in SCCs. (**C**) Schematic drawing of the left SCCs’ position in vertical view of the head.

The volunteers sitting on the rotatable chair fastened the safety belts, and wore the eyepatch which was adjusted to record the pupil movement. As shown in Fig. 7A,C, the volunteers maintained a head position with the axis of rotation perpendicular to the ground and passing through the middle point P1 of both ears. We marked the auxiliary signs on the chair railings to keep the volunteer’s head in the correct position. The chair rotated anticlockwise for 7 s with a constant angular acceleration of 30°/s^2^, and the initial rotational velocity of 0. The gyroscope recorded the real-time angular velocity of the volunteer’s head when the chair was rotating. All the volunteers firstly participated in the experiment without using the refrigerating device (control experiment), and then participated in the experiment using the refrigerating device (normal experiment). To eliminate the interference between the same volunteers in normal and control experiments, the volunteers rested for at least 30 min before the next experiment. All experiments were performed in a dark room to exclude the disturbance of light to eye movement. Besides, the volunteers used refrigerating device for 5 min in advance before the normal experiment to ensure that the temperature of the SCCs decreased and achieved the balance between heat and cold.

### Nystagmus processing

The recorded videos of eye movement were processed by MATLAB R2017b to track and locate the center of the pupil (see Fig. 6D). We discarded the first and last data of nystagmus slow phases, and removed the phases shorter than 50 ms to reduce the statistical error^21^. Besides, the first 3 s of the nystagmus was discarded because the SPV generally increases within 3 s after the head senses the change in acceleration^17^. We also removed the nystagmus data when the volunteers blinked. The SPV was calculated based on the method provided by Wu et al.^15^.

### Numerical modeling

We reconstructed the 3D geometric model of SCCs in human left ear according to the parameters provided by Ifediba et al.^22^. The geometric model of SCCs included utricle, three semicircular canals, and three cupulae (see Fig. 7B). The geometric model of SCCs was meshed by Hypermesh (version 12.0). The endolymph domain consisted of 214 k tetrahedral elements and 48 k nodes. The three cupulae domains consisted of 41 k tetrahedral elements and 9 k nodes. Additionally, the results of numerical simulation were almost the same when we refined mesh in endolymph domain with 492 k tetrahedral elements and 103 k nodes, indicating that the selected number of elements for the original model was appropriate.

The cupula is a gelatinous structure^9,23,24^. Its density is 1000 kg/m^3^^25,26^; the Young’s modulus is 5 Pa^5^; the Poisson ratio is 0.48^5,7,27^. The endolymph is similar to water^24,28–30^, with the density of 1000 kg/m^3^ and viscosity of 0.0085 Pa^7,25^. These parameters commonly represented the physical properties of the SCCs at the temperature of 37 °C. When the temperature of the SCCs dropped to 36 °C, the density and viscosity of the endolymph increased by 0.036% and 1.83% respectively based on the properties of water^31^.

We constructed the computer models including the endolymph and three cupulae in ANSYS Workbench (version 16.0) based on the method provided by Goyens et al.^9^. When the SCCs are rotating, the endolymphatic movement in the earthbound reference frame can be expressed by Navier–Stokes equations^32^:

1 $$\rho \frac{{\partial {\varvec{u}}}}{\partial t} + \rho \left( {{\varvec{u}} \cdot \nabla } \right){\varvec{u}} = - \nabla P + \mu \nabla^{2} {\varvec{u}}$$

where *ρ* is the fluid density, ${\varvec{u}}$ is the flow velocity vector, *P* is the static pressure, and *µ* is the dynamic viscosity. Besides, considering the relative reference frame moving with the walls of SCCs, the walls of endolymph are stationary. Hence, the movement of endolymph in the relative reference frame can be described by the following equations^32^:

2 $$\rho \frac{{\partial {\varvec{v}}}}{\partial t} + \rho \left( {{\varvec{v}} \cdot \nabla } \right){\varvec{v}} = - \nabla P + \mu \nabla^{2} {\varvec{v}} - 2\rho {{\varvec{\Omega}}} \times \left( {{{\varvec{\Omega}}} \times {\varvec{r}}} \right) - \rho \frac{{\partial {{\varvec{\Omega}}}}}{\partial t} \times {\varvec{r}}$$

where ${\varvec{v}}$ is the fluid velocity vector relative to the velocity of the moving reference frame, **Ω** = (0, 0, *ω*) is the angular velocity vector of the moving reference frame, and ${\varvec{r}}$ is the radial coordinate of the fluid element. Besides, the Navier equation of motion for the cupula can be written as

3 $$\rho_{s} \frac{{\partial^{2} {\varvec{d}}}}{{\partial t^{2} }} = \nabla \cdot {\varvec{\sigma}}_{{\varvec{s}}}$$

where $\rho_{s}$ is the cupular density, ***d*** is the displacement vector, and the stress tensor ${\varvec{\sigma}}_{{\varvec{s}}} \user2{ }$ is defined as below:

4 $${\varvec{\sigma}}_{{\varvec{s}}} = 2\mu {\varvec{\varepsilon}} + \lambda tr\left( {\varvec{\varepsilon}} \right){\mathbf{I}}$$

where ${\varvec{\varepsilon}}$ is the strain tensor, and $\mu$ and $\lambda$ are Lamé coefficients, which are related to Young’s modulus *E* and Poisson’s ratio *ν*, based on the following equations:

5 $$\mu = \frac{E}{{2\left( {1 + \nu } \right)}}$$

6 $$\lambda = \frac{\nu E}{{\left( {1 + \nu } \right)\left( {1 - 2\nu } \right)}}$$

Equations (3)–(6) are cited from Selva et al.^33^.

We used the Ansys Workbench (version 16.0) to simulate the rotation of SCCs at temperatures of 36 °C and 37 °C, respectively. The numerical model was loaded with the anticlockwise rotational acceleration of 30°/s^2^, and the initial rotational velocity of 0. Furthermore, we observed that the results of cupula responses were stable after 2 s in the early calculations (e.g. As shown in Fig. 4A, the maximal cupula deformation gradually increases during 0–1 s, and then tends to be approximately stable). Thus, the computational time was set as 2 s in order to reduce unnecessary calculating time. The time step was set as 0.001 s.
